# Decannulation ahead: a comprehensive diagnostic and therapeutic framework for tracheotomized neurological patients

**DOI:** 10.1186/s42466-025-00376-1

**Published:** 2025-03-17

**Authors:** Rainer Dziewas, Tobias Warnecke, Bendix Labeit, Volker Schulte, Inga Claus, Paul Muhle, Anna Brake, Lena Hollah, Anne Jung, Jonas von Itter, Sonja Suntrup-Krüger

**Affiliations:** 1 Department of Neurology and Neurorehabilitation, Klinikum Osnabrück – Academic Teaching Hospital of the University of Münster, Am Finkenhügel 1, Osnabrück, Germany; 2 Department of Neurology, Medical Faculty and University Hospital Düsseldorf, Düsseldorf, Germany; 3 Department of Neurology with Institute for Translational Neurology, University Hospital Münster, Münster, Germany

## Abstract

**Background:**

Decannulation in tracheotomized neurological patients is often complicated by severe dysphagia, which compromises airway safety and delays weaning. Additional challenges, including reduced cough strength, excessive bronchial secretions, and altered airway anatomy exacerbate weaning issues, thereby increasing morbidity and mortality. This review summarizes diagnostic procedures and therapeutic options crucial for the rehabilitation of tracheotomized patients.

**Main body:**

Key diagnostic strategies for assessing decannulation readiness focus on airway protection, airway patency, bronchial secretion management, and cough function. These are collectively introduced as the A^2^BC criteria in this review. Advanced tools such as flexible endoscopic evaluation of swallowing, endoscopic assessment of airway anatomy, measurement of cough strength, and intrathoracic pressure are essential components of a systematic evaluation. Therapeutic interventions encompass restoring physiological airflow, behavioral swallowing treatment, secretion management, and pharyngeal electrical stimulation. The proposed decannulation algorithm integrates two pathways: the “fast-track” pathway, which facilitates rapid decannulation based on relevant predictors of decannulation-success, and the “standard-track” pathway, which progressively increases cuff deflation intervals to build tolerance over time.

**Conclusion:**

Successful decannulation in neurological patients demands a multidisciplinary, patient-centered approach that combines advanced diagnostics, targeted therapies, and structured management pathways. The proposed algorithm integrates fast-track and standard-track pathways, balancing rapid diagnostics with gradual weaning strategies. This framework promotes flexibility, enabling clinicians to tailor interventions to individual patient needs while maintaining safety and optimizing outcomes.

## Introduction

The tracheotomy, particularly the minimally invasive dilatational approach, is a standard procedure in most intensive care units (ICU) [1]. It is performed in 10–15% of patients in mixed ICUs [2] and in 15–47% of patients in a neurocritical care setting [3, 4]. Especially in patients with a leading neurological diagnosis (e.g. stroke, traumatic brain injury, immunoneuropathies), but also in patients with severe critical illness polyneuropathy (CIP) and critical illness myopathy (CIM), weaning from the tracheostomy cannula is often impossible during the acute stage of treatment [5–7], so that these patients are transferred to early rehabilitation while still cannulated. Accordingly, in a multicenter observational study on the rehabilitation process of survivors of acute neurological diseases, 41.5% of patients had a tracheostomy requiring suctioning [8]. In a second study, 53.2% of patients with traumatic brain injury and 22.5% of stroke victims were tracheotomized at the beginning of rehabilitation [9]. In most studies, the subsequent decannulation rates during rehabilitation, which often lasts several months, are between approximately 40 and 60% [8, 10–12]. The therapeutic goal of weaning from the tracheostomy cannula is of overriding prognostic importance, as the mortality rate of patients who have been permanently cannulated is extraordinarily high. For example, in a long-term observational study in which over 1000 early rehabilitation patients were included, about 50% of the tracheotomized patients had died within one year, while the mortality rate in the group of non-tracheotomized patients was about 10% [8, 12].

One of the key reasons why neurological patients are prone to delayed or failed decannulation is severe dysphagia with related impaired airway safety [13–15]. Other less disease-specific factors that complicate the weaning process consist of reduced cough strength, excessive amount of bronchial secretions and impaired airway anatomy [16]. The diversity of these parameters and assigned medical topics underlines that the care of tracheotomized patients is one of the classic team tasks in modern medicine [17]. Depending on the local conditions, the multi-professional team includes various specialists such as intensive care and rehabilitation physicians, neurologists, ENT doctors, phoniatricians, respiratory therapists, speech and language pathologists and intensive care nurses [18]. Although there are no prospective randomized trials on this topic so far, and all recommendations are therefore based on weak evidence [19], numerous studies with different designs and some meta-analyses suggest that this interdisciplinary approach improves the conditions for rapid and safe tracheostomy tube removal [18, 20–28]. Therefore, in a current guideline of the American Association for Respiratory Care (AARC), the implementation of multi-professional teams is therefore recommended as a third pillar in addition to tracheostomy bundles and decannulation protocols adapted to the respective setting [27].

This review provides an up-to-date summary of diagnostic procedures and therapeutic options relevant to the rehabilitation of tracheotomized patients. In its final part an algorithm for decannulation management is presented, which combines the diagnostic and therapeutic interventions explained before and distinguishes between a pathway for rapid decannulation and a slower, conventional approach.

## Diagnostics

### Clinical procedures

According to a recent guideline, the clinical swallow examination (CSE) is usually performed as the first diagnostic step in tracheotomized patients [29]. After deflating the cannula’s cuff and careful subglottic and oropharyngeal suctioning, physiological air flow through the upper airway is restored by closing the cannula or using a speaking valve. This is followed by the CSE, which is based on standard procedures, and, among others, looks for clinical signs of penetration and aspiration of saliva and administered food boluses. In accordance with the low reliability of the CSE for determining swallowing safety, the sensitivity of this method in comparison to gold standard FEES is low [30].

As an additional clinical instrument, the Evans Blue dye Test (EBT) and the modified Evans Blue dye Test (mEBT) have been introduced into practice [31]. To perform the EBT, the cannula’s cuff is first deflated and subglottic and pharyngeal secretion is carefully suctioned. The patient then receives a few drops of food coloring directly on the tongue (EBT) or is given small amounts of colored liquid or other food consistencies to swallow (mEBT). Thereafter, subglottic suctioning is repeated and if colored secretion (EBT) or colored liquid/food (mEBT) is detected, a high risk of aspiration is suspected. According to several studies [31–34] and a meta-analysis [35], this method has insufficient sensitivity. Only two studies employing repeated suctioning suggest an acceptable accuracy of the (m)EBT [36, 37]. Therefore, a negative (m)EBT is of no diagnostic value, but a positive (m)EBT is indicative of a substantial risk of aspiration in tracheotomized patients. In view of this scientific context, the (m)EBT should be classified as a screening instrument that can only be used to follow-up instrumental evaluation. The exclusive use of the (m)EBT to assess readiness for decannulation is not recommended [29, 38].

### Flexible endoscopic evaluation of swallowing

During flexible endoscopic evaluation of swallowing (FEES), a flexible naso-pharyngo-laryngoscope is introduced transnasally into the pharynx for direct visualization of the swallowing act. FEES aims at identifying pathological movement patterns, evaluating the effectiveness and safety of the swallow process, recommending appropriate food consistencies as well as special diets or swallowing techniques and delineating potential phenotypes and etiologies related to the observed swallowing impairment [39, 40]. Available data indicate that FEES is a well-tolerated and safe examination [41–44]. In the ICU and neurorehabilitation facilities the essential practical advantages of FEES are that the examination can be performed at the bedside and also patients with highly restricted motor functions as well as bedridden or uncooperative patients can be examined, repeated follow-up examinations are safely possible and saliva management can be assessed directly [45, 46].

**Fig. 1:**
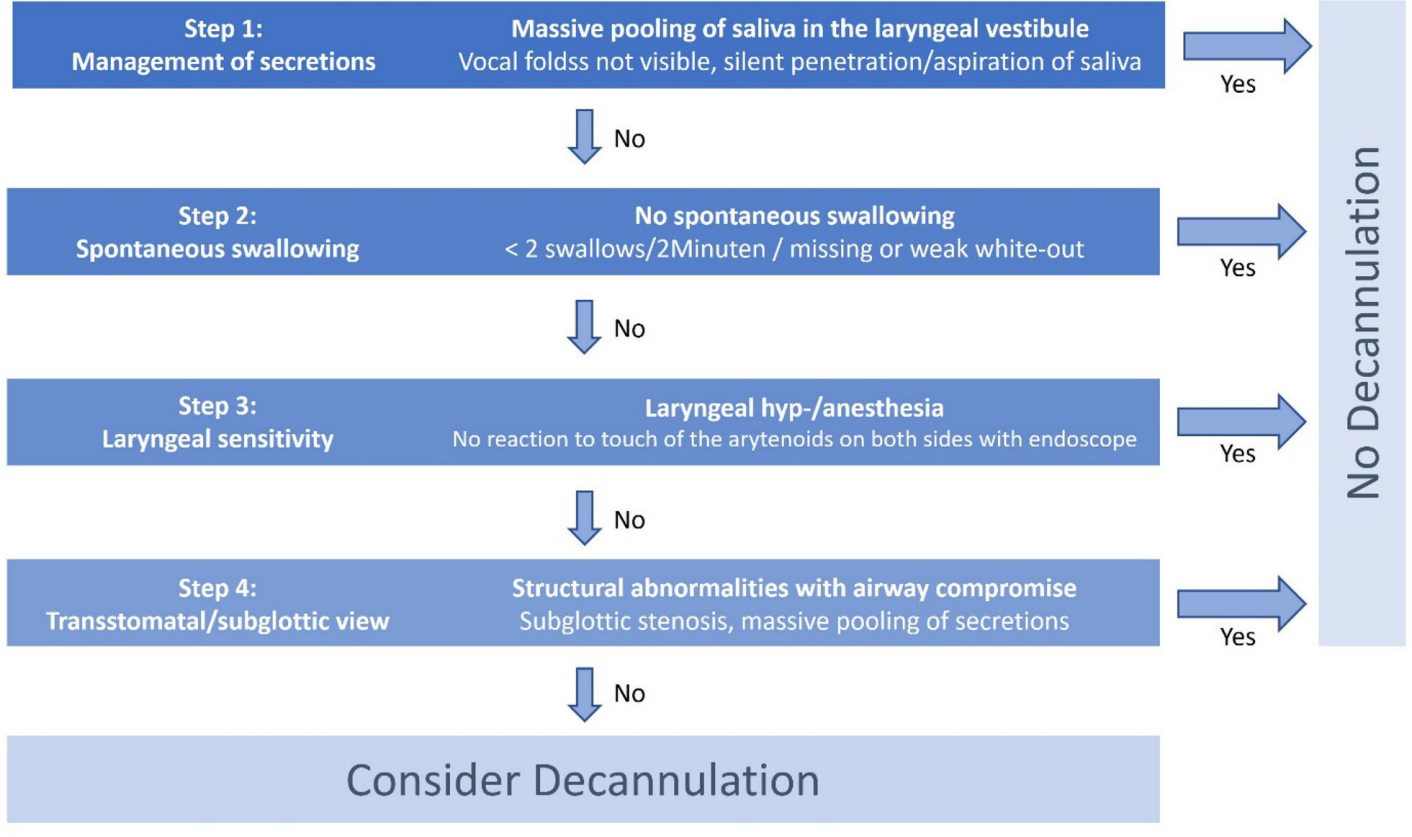
Standardized Endoscopic Swallowing Evaluation for Tracheostomy Decannulation (SESETD) protocol (modified with permission from [48, 49]

**Fig. 2:**
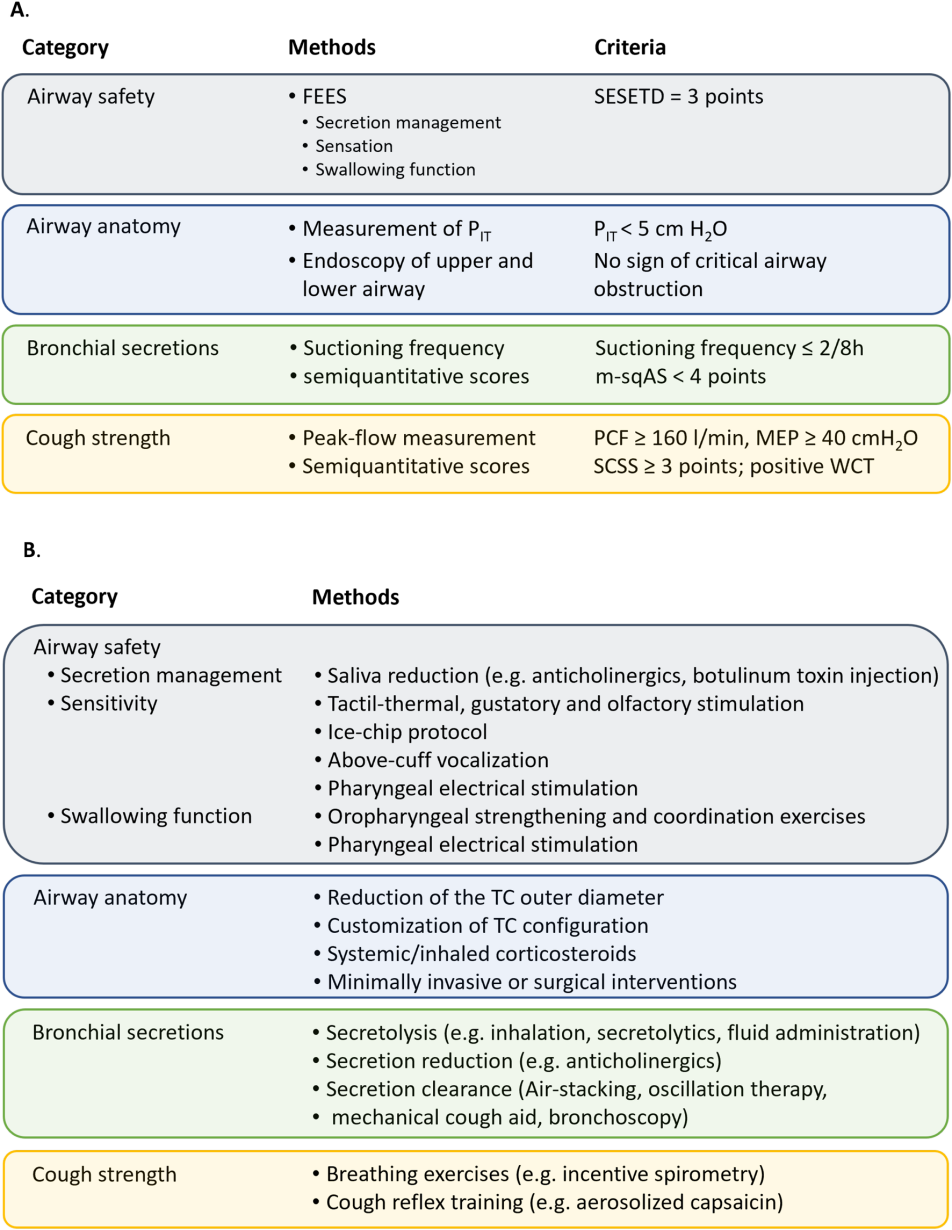
**A** Multimodal diagnostics and related thresholds for the assessment of tracheotomized patients according to the A^2^BC criteria (A = Airway safety & airway anatomy, B = bronchial secretion, C = cough strength; FEES = fiberoptic endoscopic evaluation of swallowing; MEP = maximum expiratory pressure; m-sqAS = modified semiquantitiative airway score; PCF = peak cough flow; P_It_ = intrathoracic pressure; SCSS = semiquantitative cough strength score; SESETD = standardized endoscopic swallowing evaluation for tracheostomy decannulation; WCT = White-card-test). **B** Multimodal treatment of tracheotomized patients targeting A^2^BC problems (TC = Tracheal cannula)

As per a recent guideline, in tracheotomized patients FEES should specifically assess airway safety by evaluating the parameters “secretion management”, “spontaneous swallowing rate”, and “laryngeal sensitivity” [29]. To increase the reliability and reproducibility of the endoscopic examination, the **S**tandardized **E**ndoscopic **S**wallowing **E**valuation for **T**racheostomy **D**ecannulation (SESETD) protocol has been developed and validated [47–49] (see Figs. 1 and 2A). According to this protocol, after suctioning pharyngeal and subglottic secretions and deflating the tracheal cuff, the extent and localization of salivary retentions are assessed, and the spontaneous swallowing frequency is observed. To get a realistic impression of how and to what extent the patient manages his secretions, this part of the examination requires several minutes. In case there is massive pooling of saliva in the hypopharynx with concomitant penetration and aspiration, the tracheal cannula should not be removed. If there is no issue with pharyngeal secretions but the swallowing frequency is below one per minute or due to a missing white-out a severe pharyngeal palsy is suspected, the tracheal cannula should also remain in place. The third step of the SESETD protocol involves testing laryngeal sensitivity by gently touching the aryepiglottic region with the tip of the endoscope. If no motor reaction occurs during this procedure, severe peripheral sensory disruption must be assumed and decannulation should consequently be delayed. In case the patient coughs, swallows or shows other kinds of reflexive movements, such as a laryngeal adductor reflex or pharyngeal wall contraction, the cannula may be removed. The protocol’s final step consists of transstomatal endoscopy. Here, the endoscope is briefly inserted through the stoma, flexed upward to visualize the subglottic structures and downward to inspect the lower trachea in order to ensure that there are no structural abnormalities compromising the airway and precluding decannulation [32, 50–52]. In patients who successfully pass all four steps of the SESETD protocol, the tracheal cannula may be permanently removed, and the tracheostomy subsequently closed. Alternatively, a tracheostoma retainer may be used for 24–48 h, or the patient may be monitored using a capped and unblocked cannula.

### Evaluation of cough and bronchial secretions

In addition to the swallowing function, strength and effectiveness with regards to secretion removal of the cough and the type and amount of bronchial secretion should be systematically evaluated [53]. The peak cough flow (PCF) and the maximum expiratory pressure (MEP) generated during coughing can be quantified with a peak flow meter. A PCF of 160 l/min or a MEP of 40 cmH_2_O are usually mentioned in the literature as indicators for safe decannulation [54–56] (Fig. 2A).

Alternatively, qualitative clinical cough scores can be used. For example, the semiquantitative cough strength score (SCSS) rates the cough strength on a 6-point scale as 0 = no cough, 1 = air burst but no audible cough, 2 = barely audible cough, 3 = audible cough, 4 = stronger cough, 5 = multiple strong coughs [57]. In two prospective observational studies recruiting 91 and 186 endotracheal intubated patients, respectively, an SCSS of ≥ 3 was identified as the threshold for extubation [57, 58]. In addition, the SCSS correlated with the PCF measurement (Fig. 2A). As a simpler, binary score, the so-called white card test (WCT) has been proposed, which tested whether patients expectorate secretions against a “white card” held 1–2 cm in front of the end of the tube [57]. This test was developed for patients with an orotracheal tube but seems to be suitable for tracheotomized patients as well (Fig. 2A). As demonstrated in a recent study, when comparing PCF measurements with the WCT, the quantitative method appears to more precisely predict the successful removal of the artificial airway [59].

The type and amount of bronchial secretion should also be evaluated semi-quantitatively (with the cuff inflated or deflated depending on the clinical condition) [60]. In a recent study, the suctioning frequency was used as an easy-to-determine parameter and a frequency of no more than 2 suctions every 8 h was considered indicative of readiness for decannulation [61]. In addition to the suctioning frequency, a more differentiated method also assesses the amount of secretion via suctioning passes. The secretion characteristics are described according to its viscosity and color [62]. In addition, the authors also took into account the parameters cough and gag reflex, so that this score enables a relatively comprehensive description of airway protection. A modified semiquantitative airway score (m-sqAS) was a significant predictor of extubation failure in intubated stroke patients in two other prospective observational studies [63, 64] (Table 1). In the first study, the successfully extubated patients had an m-sqAS of 4 ± 4 points compared to 8 ± 3 points in the reintubated patient group [63]; in the second study the average values were 3 ± 2 versus 5 ± 3 points [64], so that a threshold of < 4 points seems reasonable to predict successful extubation.

**Table 1:** Modified semiquantitative airway score (m-sqAS; adopted from [63, 64])

Points	Spontaneous cough	Gag	Sputum quantity	Suctioning frequency	Sputum viscosity	Sputum character
0	Vigorous	Vigorous	None	> 3 h	Watery	Clear
1	Moderate	Moderate	1 Pass	2–3 h	Frothy	Tan/yellow
2	Weak	Weak	2 Passes	1–2 h	Thick	–
3	None	None	3 Passes	< 1 h	–	–

### Evaluation of airway anatomy

As a complication of a tracheotomy, clinically relevant stenosis with a lumen narrowing of more than 20% is to be expected in 10–20% of patients [65]. In addition to fixed stenosis due to scarring, cartilage ring fractures, granulation tissue or tissue swelling, flexible airflow dependent stenosis due to tracheomalacia with resulting tracheal instability can also be observed. Therefore, prior to definitive decannulation, a careful evaluation of the airway should be carried out. If the subglottic, transstomatal endoscopy does not allow for firm conclusions, additional translaryngeal endoscopy is recommended for the assessment of the laryngotracheal junction [66] (Fig. 2A).

Tracheal tube manometry providing measures for the intrathoracic pressure (P_IT_) may be helpful to objectively guide recommendations for speaking valve use, capping, and changing tracheostomy tubes. Measurements are done with an unblocked cannula in place, which is equipped with either a speaking valve or a cap [67]. Technically, this measurement is carried out via a manometer that is connected between the cap/speaking valve and the tracheostomy cannula [68]. Pressure values below 5 cm H_2_O suggest unimpaired breathing. For values between 5 and 10 cm H_2_O, short-term breathing through the upper airways is usually feasible under continuous patient observation. Values above 10 cm H_2_O indicate critically increased airway resistance [67, 68] (Fig. 2A). In the latter scenario, an endoscopic evaluation of the airway anatomy should be carried out. If necessary, the cannula should be changed with an adaptation of the tracheostomy cannula configuration (e.g. change to a cannula with a smaller outer diameter or use of a fenestrated cannula [68].

### Decannulation criteria

The literature describes numerous decannulation criteria and scores, each tailored to different patient populations. Most of these approaches use an observation period as key component, which is used to test whether the patient remains respiratory stable over a period of a defined length (e.g. 24–72 h) without airway protection (e.g. with an unblocked and closed tracheostomy tube). Additionally, clinically or endoscopically determined parameters are considered, focusing primarily on the level of secretions, cough strength, swallowing function, and airway anatomy [21, 50, 69–75]. Enrichi and colleagues propose several parameters for decannulation based on the results of a meta-analysis and a subsequent prospective observational study. The identified decannulation parameters include ‘72-h tracheostomy tube occlusion,’ ‘ endoscopically confirmed airway patency,’ ‘ no aspiration during endoscopic swallowing assessment (Penetration-Aspiration Scale ≤ 5),’ and ‘ negative mEBT,’ demonstrated by three instances of subglottic suctioning over a 12-h period. Conversely, criteria such as voluntary or reflexive coughing and the volume of tracheal secretions requiring suctioning were found to be less significant in determining the validity of decannulation decisions [74].

The selection of decannulation criteria is largely determined by the clinical setting and patient population. For typical early neurorehabilitation patients treated in an intermediate care or a related environment, a gradual weaning strategy combined with a repeated, multimodal assessment following one of the aforementioned structured approaches appears to be appropriate. In contrast, for patients where the question of decannulation arises during acute care, such as in the intensive care unit, alternative criteria are considered more suitable. For instance, Cohen et al. demonstrated in a retrospective case–control study that, in the intensive care unit, decannulation immediately following endoscopic evaluation was associated with fewer recannulations, a shorter period of unassisted spontaneous breathing before decannulation, and a reduced hospital length of stay post-decannulation compared to a protracted decannulation management [76]. A similar result was found in a multicenter, randomized intervention study that included 330 tracheotomized, ventilator-weaned ICU patients [61]. The control condition involved decannulation when patients tolerated tracheostomy tube capping for 24 h. In the intervention arm, decannulation was done as soon as the suctioning frequency over a 24-h period was no higher than 2 times per 8 h. Patients in the intervention arm were decannulated significantly earlier (6 versus 13 days), had fewer bronchopulmonary infections (23% versus 39.1%), and shorter hospital stays (23 versus 37 days) after randomization compared to patients of the control group [61]. Recannulation rates were comparable in both groups, with 2.4% in the study arm and 5.6% in the control condition. This study demonstrates that, at least under certain conditions, the criterion of tolerating tracheostomy tube occlusion over a longer observation period (≥ 24 h) is associated with unnecessary delays in decannulation and increased complications.

### A^2^BC criteria

Based on the evidence summarized above, the A^2^BC criteria are introduced to link each relevant clinical domain (**a**irway safety, **a**irway anatomy, **b**ronchial secretions, **c**ough strength) with specific quantitative or qualitative thresholds indicating readiness for decannulation (Fig. 2A). With regards to airway safety the SESETD algorithm is recommended and patients passing all 4 steps are considered to have a safe airway. For assessing airway anatomy subglottic and, where needed, translaryngeal endoscopy should be done. During the weaning process, measurement of P_IT_ may help to adjust the configuration of the tracheal cannula (TC). The amount of bronchial secretions may be scored with the m-sqAS (threshold < 4 points) or the suctioning frequency, whereas for the latter a threshold of ≤ 2/8 h may be used. Finally, cough strength is recommended to be measured directly by spirometry (thresholds PCF ≥ 160 l/min, MEP ≥ 40 cmH_2_0), alternatively the SCSS (≥ 3points) or the WCT may be employed.

## Treatment

This section summarizes various therapeutic options to improve swallowing function, airway protection, and secretion management in tracheotomized patients, thereby targeting potential A^2^BC issues (see also Fig. 2B).

### Establishing a physiological airflow through the upper airways

**Fig. 3:**
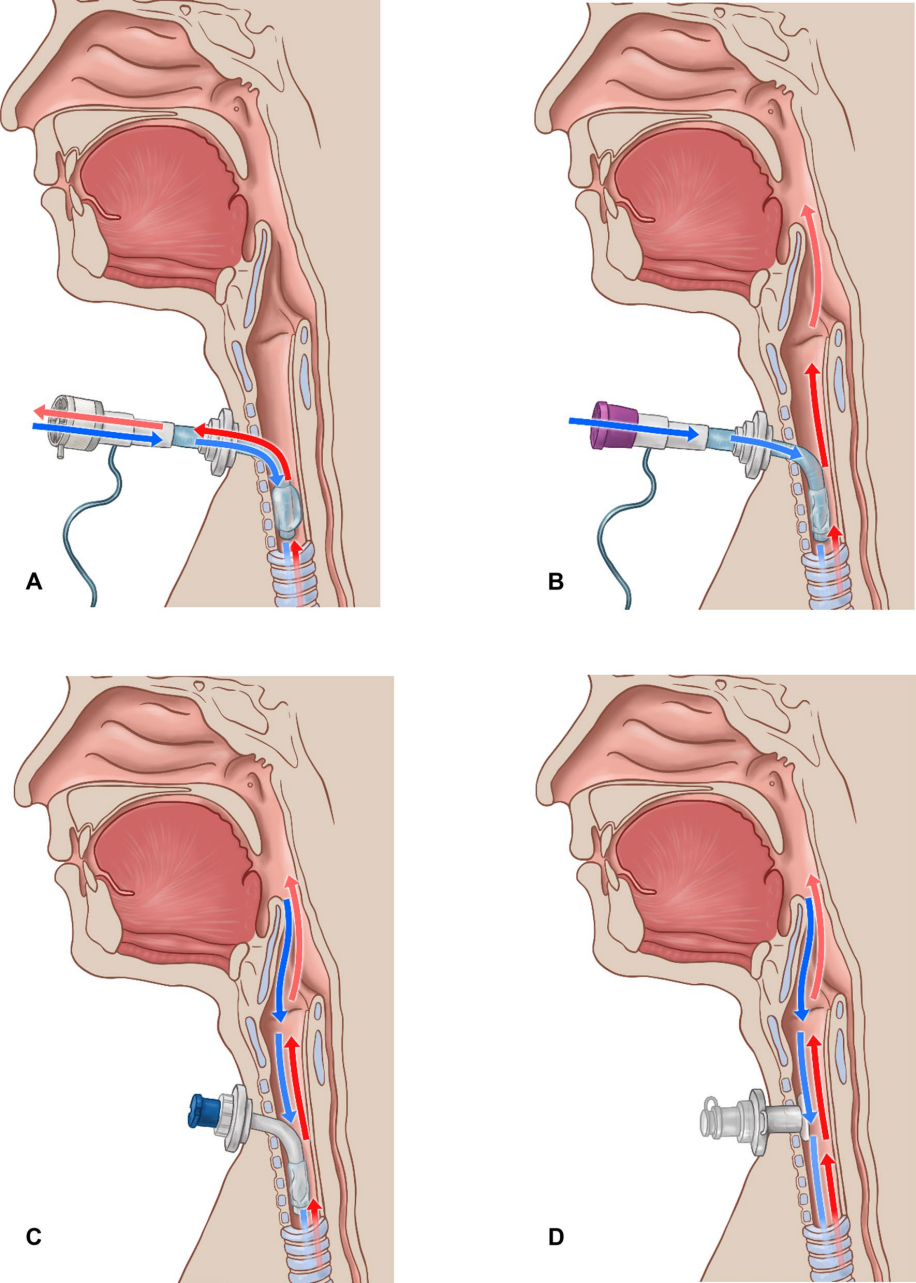
Restoring a physiological airflow through the upper airway during the process of tracheal cannula weaning; **A**: blocked tracheal cannula (TC), **B**: deflated cuff, TC closed with a speaking valve, **C**: TC closed with a cap; **D**: use of a tracheostomy retainer (reproduced with permission; © Michael Hoffmann, medicalgraphics, cologne)

Especially when a gradual weaning from the tracheostomy tube is required, regular and progressively longer periods of cuff deflation with simultaneous closure of the tracheostomy tube or the use of a speaking valve should be applied [21, 68, 77] (Fig. 3B and C). The restoration of physiological airflow is likely to contribute to the recovery of pharyngeal and laryngeal sensation, resulting in improved secretion management [78]. In a proof-of-principle study recruiting 20 tracheotomized stroke patients, the authors showed that while capping of the tracheal cannula did not induce any changes in swallowing mechanics, swallowing safety improved resulting in lower Penetration-Aspiration-Scale scores [79]. Furthermore, physiological airflow allows for smelling and phonation, and through the gradual increase in airway resistance, it strengthens the respiratory muscles [16].

Before moving on to longer periods of cuff deflation, it is often necessary to switch the tracheal cannula to one with a smaller outer diameter to reduce airway resistance [67, 75]. Using fenestrated cannulas can also promote airflow through the larynx and allow for phonation [80, 81]. Lastly, as a precaution before complete decannulation, a placeholder can be inserted to keep the option of recannulation open, at least temporarily (Fig. 3D) [82].

### Above-cuff vocalization

Above-cuff vocalization (ACV) is a technique that enables tracheostomized patients with inflated cuffs to speak by directing a controlled airflow through the subglottic suction port, allowing air to pass over the vocal cords [83]. This method not only facilitates communication but also provides sensory stimulation to the laryngeal mucosa, which may enhance swallowing function and aid in the weaning process [83]. Studies have demonstrated that ACV is feasible and safe, contributing to earlier speech initiation in critically ill patients [84]. However, its implementation requires careful patient selection and monitoring to prevent potential complications [85]. Further research is needed to establish standardized protocols and to fully understand the benefits of ACV in tracheostomy weaning.

### Behavioral swallowing therapy

In the early stages of neurological rehabilitation for severely impaired patients, the main goal of dysphagia therapy is to stimulate swallowing and prevent or resolve a nil-per-os (NPO) status [86]. Encouraging swallowing, even of small boluses, helps break the vicious cycle often seen in tracheotomized patients, where reduced swallowing activity worsens the condition. On the one hand, less frequent swallowing leads to the buildup of thick secretions in the hypopharynx that coat the mucosa. On the other hand, this patient group frequently experiences pharyngeal and laryngeal swelling, partly due to poor lymphatic drainage from the lack of muscle activity in the pharynx caused by reduced swallowing [86]. Both of these issues impair pharyngeal and laryngeal sensitivity, with known negative consequences for airway safety.

In clinical practice, swallowing exercises and maneuvers are routinely used in the treatment of tracheotomized patients [86]. The so-called restorative methods aim to restore impaired swallowing function or enhance residual functionality. These methods include preparatory stimulation techniques (e.g., thermal stimulation), mobilization techniques (e.g., tongue pressure against resistance), and autonomous movement exercises (e.g., Shaker exercise, Masako maneuver, Expiratory Muscle Strength Training (EMST)) [29, 87]. In contrast, compensatory methods are used during the swallow to enable effective and safe deglutition despite functional impairments. A distinction is made between postural maneuvers (e.g. chin-tuck or head-turn maneuvers) and special swallowing techniques (e.g. Mendelsohn maneuvers, supraglottic swallowing) [88]. Particularly for patients with a preserved ability to cooperate, the supplementary use of biofeedback techniques during swallowing therapy may be considered. Surface EMG [89], submental ultrasound [90], and FEES [91] are suggested in the literature as technical alternatives.

### The ice chip protocol

The Ice Chip Protocol described by Susan Langmore represents a classic hybrid of diagnostic and therapeutic procedures and was explicitly developed for the treatment of severely dysphagic, tracheotomized patients [92]. In these patients, ice-chips are particularly suitable since (i) the cold sensation acts as a strong sensory trigger that boosts swallowing activity, (ii) due to their solid consistency ice-chips can be better controlled in the mouth than liquid boluses, which more likely spill into the pharynx, (iii) they can be given in variable but defined amounts depending on the patient’ s swallowing abilities (an ice chip has a volume of approximately 1 ml), and (iv) the patient is reasonably safe if aspiration occurs [93–95]. Ice-chips should first be used within FEES to assess the effects of the intervention on parameters such as swallowing frequency and secretion status and to determine the appropriate bolus size for the patient. Subsequently, ice chips can be regularly included into swallowing therapy, often serving as a transition between “nil-per-os” status and the introduction of regular food textures, such as semisolids or liquid boluses [94].

### Secretion management

Depending on the clinical context, secretion management aims at reducing secretion, promoting secretolysis, and/or improving its mobilization and expectoration. For reducing secretions, systemically active anticholinergic drugs are available, with glycopyrronium bromide having the advantage over other anticholinergics like scopolamine, as it doesn’ t cross the blood–brain barrier, leading to fewer neurocognitive and psychiatric side effects [16]. In cases of significantly impaired saliva management with notable residues in the pharynx, injection of botulinum toxin into the salivary glands is recommended [96]. For thick and/or dry secretions, continuous or intermittent humidification of the breathing air should be performed, fluid intake should be adjusted, and the use of mucolytics, such as hypertonic saline inhalation, should be considered [16].

To mobilize and remove secretions, both invasive procedures like endotracheal suctioning and bronchoscopy, and non-invasive techniques, including general physio- or respiratory therapeutic approaches (mobilization, breathing exercises, positioning) and specific techniques such as oscillation therapy and mechanical cough aids, should be employed [66].

Particularly in patients with respiratory muscle weakness, the cough can be strengthened by regular training with incentive spirometry [66]. Additionally, inhalation therapy with aerosolized capsaicin may also be used to enhance the cough reflex [97–99].

### Pharyngeal electrical stimulation

Pharyngeal Electrical Stimulation (PES) delivers electrical current to the pharyngeal mucosa via a specially designed feeding tube equipped with a pair of bipolar ring electrodes. A typical PES treatment cycle consists of 10 min of stimulation administered on three consecutive days. The stimulation intensity is individually adjusted for each session to align with the patient’ s sensation and tolerance thresholds. Physiologically, PES operates on two levels. On the cortical level, PES enhances the reorganization of the swallow-related motor cortex and facilitates activation of corticobulbar pathways [100, 101]. On the level of the peripheral nervous system, PES directly induces the release of Substance P from peripheral nerve endings, thereby amplifying afferent sensory input into the swallowing network [102–105].

In clinical practice, PES has been successfully adopted to treat dysphagia in tracheotomized patients. The PHAST-TRAC trial (PHAryngeal electrical Stimulation for early decannulation in TRACheotomised patients with neurogenic dysphagia after stroke) randomized stroke patients with a tracheal cannula and severe dysphagia precluding decannulation to one cycle of PES or sham stimulation [106]. Following the intervention, nearly 50% of patients in the treatment group were ready for decannulation, while spontaneous recovery of swallowing function allowing for a removal of the tracheal cannula occurred only in 9% of patients in the sham group. This therapeutic effect was strikingly consistent with findings from a prior single-center trial [107].

The subsequently conducted PHADER registry (The PHAryngeal electrical stimulation for treatment of neurogenic Dysphagia European Registry), a prospective phase IV trial, documented the use of PES in 245 patients assigned to different diagnostic groups [108]. In all study groups dysphagia severity improved continuously across the observational period until day 92. Two-thirds of the 99 tracheotomized patients included in PHADER were decannulated after treatment with PES, the majority within 9 days after the first treatment had been applied [108, 109]. In addition to these larger trials, PES has been successfully applied in smaller randomized and observational studies and has been featured in case reports, particularly for addressing severe dysphagia in ICU or post-ICU settings [110–118].

### Airway stenoses

Airway stenoses are a common obstacle to decannulation [50]. In a large prospective observational study recruiting 673 tracheotomized patients over a 12-month period in two rehabilitation facilities, critical airway stenoses preventing removal of the TC were found in around 4% of patients [119]. These stenoses can result from various conditions: pharyngeal narrowing (due to edema, flaccid or spastic pharyngeal and tongue muscles), space-occupying lesions of the pharyngolaryngeal structures, laryngeal edema, recurrent laryngeal nerve paralysis with medialization of the vocal folds, subglottic stenoses, excessive tracheal granulation tissue, instability of the pars membranacea (most commonly associated with respiratory overload), as well as tracheomalacia or tracheobronchomalacia [120]. The most important treatment strategy consists of optimizing the tracheal cannula configuration to minimize tissue damage [16]. If there is profound edema, corticosteroids may be used either systemically or by inhalation [121]. Minimally invasive procedures, such as endoscopic removal of granulation tissue, or surgical interventions, such as arytenoidectomy, transverse cordotomy or resection of tracheal stenoses, are particularly considered for fixed and chronic conditions [122].

## Decannulation management algorithm

Although diagnostic and therapeutic interventions in decannulation management are highly individualized and must continuously adapt to unforeseen changes in a patient’ s condition, the process should nonetheless adhere to a comprehensive overarching framework. This section, therefore, introduces an algorithm for decannulation management in ventilator-weaned, tracheotomized patients, which systematically integrates the previously described diagnostic and therapeutic components.

**Fig. 4:**
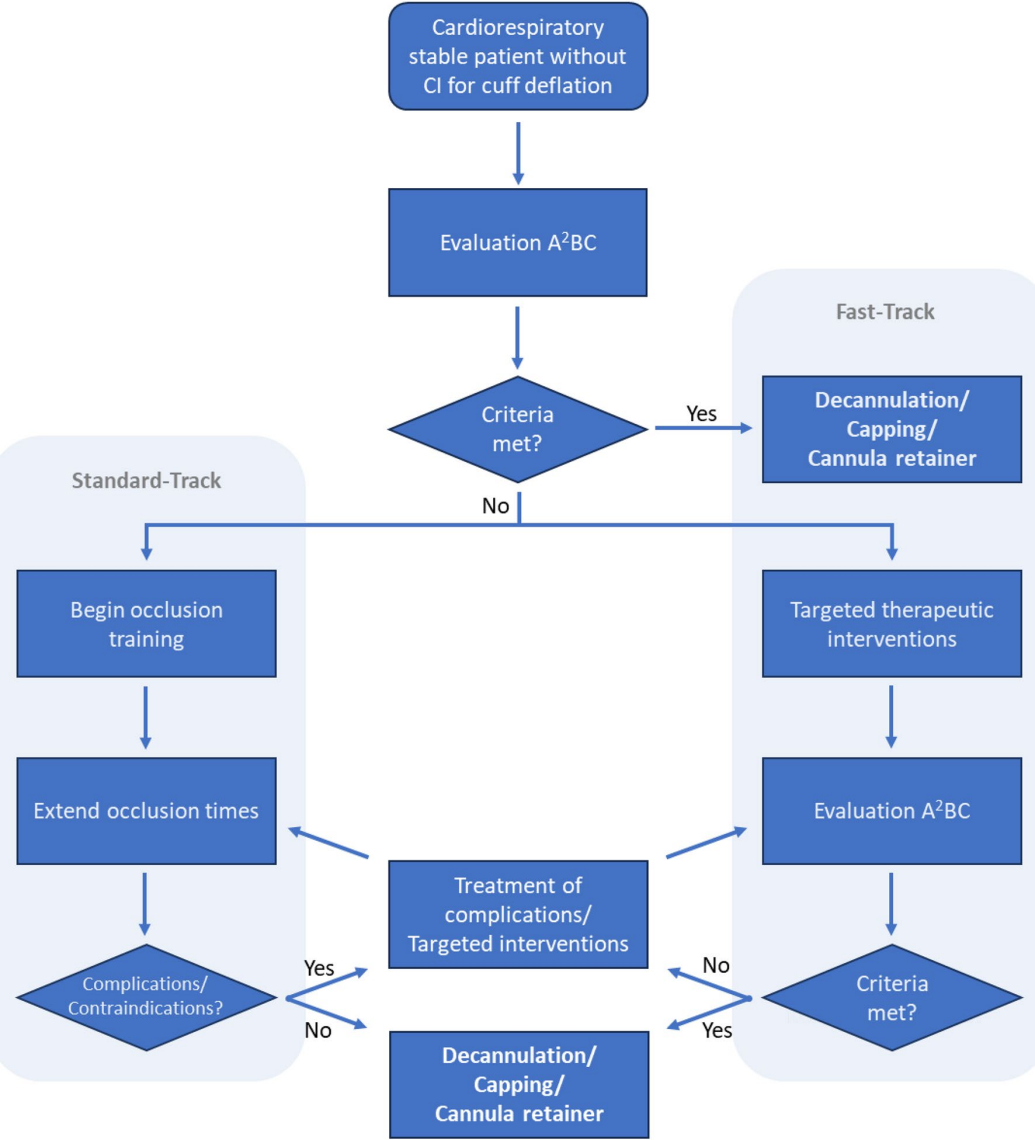
Decannulation management algorithm distinguishing between a fast-track and a standard-track pathway (CI = contraindication)

As shown in Fig. 4, the process begins by assessing whether contraindications exist for further patient evaluation, including cuff deflation. These contraindications include permanent respirator dependency, significantly reduced general condition, acute infections—particularly bronchopulmonary infections, and frequent gastric regurgitation or vomiting with subsequent penetration and aspiration [16] (Table 2A).

**Table 2:** Contraindications for cuff deflation (A) and for the extension of occlusion times (B) (modified from [16])

*A Contraindications for (initial) cuff deflation*
Permanent ventilation requirement with need for increased PEEP
Unstable general condition
Acute (bronchopulmonary) infections
Severe gastric regurgitations/frequent vomiting
*B Contraindications for extension of occlusion times*
Unstable respiratory functions with unblocked/closed cannula
∙ Tachy-/ bradypnea
∙ Desaturation episodes
∙ CO_2_ retention
∙ Airway obstructions with stridor
∙ Intrathoracic pressure > 5–10 cm H_2_O
High suctioning frequency, abundant bronchial secretion
Respiratory tract infection

If there are no contraindications present, the A^2^BC criteria (Fig. 2A) are evaluated as part of the so called *fast-track pathway* of the algorithm using the previously described diagnostic methods. With the help of the above parameters, airway protection, airway patency, type and amount of bronchial secretions, and cough function are assessed. If this evaluation does not raise any concerns, the patient may be decannulated right away or undergo a preparation period with an uncuffed and closed tracheostomy tube or a placeholder. If decannulation is not yet feasible based on the A^2^BC assessment, a targeted therapy addressing the identified key issues should be initiated (Fig. 2B). The success of the therapy should be re-evaluated at defined intervals. In the event of successful intervention, the tracheal tube can then be removed. Otherwise, further targeted interventions should be planned based on the current assessments.

Cuff deflation trials aimed at restoring physiological airflow through the upper airway should be integrated into the therapeutic concept early on. As part of the *standard-track pathway*, deflation times are progressively increased following a fixed schedule (for example: a few minutes, 30 min, 2 sessions of 1 h, 2 sessions of 2 h, 2 sessions of 4 h, 12 h, and finally 24 h) [16]. During this gradual weaning process, the patient is continuously monitored for contraindications to further extension of the deflation periods (Table 2B). If the patient stays stable for 24 to 48 h with an unblocked and closed tracheal cannula, final decannulation can be performed after airway patency has been confirmed via endoscopy. Importantly, once the clinical observation period is successfully completed, fulfilling the A^2^BC criteria is no longer required [88].

In clinical practice, the two ideal–typical treatment pathways are usually combined, particularly for patients who cannot be rapidly weaned off the tracheal cannula. The more comprehensive diagnostics included in the fast-track pathway help determine the most appropriate therapy for each individual patient. The gradual extension of cuff deflation intervals, as outlined in the standard-track pathway, serves two purposes. First, this strategy allows the clinicians to monitor whether the patient tolerates an unblocked airway, second, cuff deflation is itself an integral part of the treatment. The final decision to decannulate can then be based either on the interim fulfillment of the A^2^BC criteria or witnessing a complication-free episode of a 24–48 h cuff deflation trial.

## Conclusions

This article provides a detailed review of decannulation management in tracheotomized neurological patients, addressing the importance of a multidimensional approach and advanced diagnostics, including FEES and endoscopic airway assessment. The A^2^BC criteria, evaluating airway protection and airway patency, bronchial secretions, and cough function, are central to determining readiness for decannulation. Therapeutic interventions such as airway resistance training, behavioral swallowing interventions, and pharyngeal electrical stimulation are emphasized. The proposed algorithm integrates fast-track and standard-track pathways, balancing rapid diagnostics with gradual weaning strategies. This concept aims to enhance decannulation success rates and improve long-term outcomes in neurorehabilitation settings.

## Data Availability

Not applicable.

## Abbreviations

A^2^BC: Airway safety, airway anatomy, bronchial secretions, cough function
CIM: Critical illness myopathy
CIP: Critical illness polyneuropathy
CSE: Clinical swallow examination
(m)EBT: (Modified) Evans blue dye test
FEES: Flexible endoscopic evaluation of swallowing
ICU: Intensive care unit
MEP: Maximum expiratory pressure
m-sqAS: Modified semiquantitative airway score
PCF: Peak cough flow
PES: Pharyngeal electrical stimulation
PHADER: Pharyngeal electrical stimulation for treatment of neurogenic dysphagia european registry
PHAST-TRAC: Pharyngeal electrical stimulation for early decannulation in tracheotomised patients with neurogenic dysphagia after stroke
P_IT_: Intrathoracic pressure
SCSS: Semiquantitative cough strength score
SESETD: Standardized endoscopic swallowing evaluation for tracheostomy decannulation
TC: Tracheal cannula
WCT: White-card-test

## References

[1] Zuercher, P., Moret, C. S., Dziewas, R., & Schefold, J. C. (2019). Dysphagia in the intensive care unit: Epidemiology, mechanisms, and clinical management. *Critical Care (London, England),**23*(1), 103. 30922363 10.1186/s13054-019-2400-2 PMC6438038

[2] Abe, T., Madotto, F., Pham, T., Nagata, I., Uchida, M., Tamiya, N., Kurahashi, K., Bellani, G., & Laffey, J. G. (2018). Epidemiology and patterns of tracheostomy practice in patients with acute respiratory distress syndrome in ICUs across 50 countries. *Critical Care (London, England),**22*(1), 195. 30115127 10.1186/s13054-018-2126-6 PMC6097245

[3] Krishnamoorthy, V., Hough, C. L., Vavilala, M. S., Komisarow, J., Chaikittisilpa, N., Lele, A. V., Raghunathan, K., & Creutzfeldt, C. J. (2019). Tracheostomy after severe acute brain injury: Trends and variability in the USA. *Neurocritical Care,**30*(3), 546–554. 30919303 10.1007/s12028-019-00697-5 PMC6582655

[4] Pelosi, P., Ferguson, N. D., Frutos-Vivar, F., Anzueto, A., Putensen, C., Raymondos, K., Apezteguia, C., Desmery, P., Hurtado, J., Abroug, F., et al. (2011). Management and outcome of mechanically ventilated neurologic patients. *Critical Care Medicine,**39*(6), 1482–1492. 21378554 10.1097/CCM.0b013e31821209a8

[5] Schneider, H., Hertel, F., Kuhn, M., Ragaller, M., Gottschlich, B., Trabitzsch, A., Dengl, M., Neudert, M., Reichmann, H., & Wopking, S. (2017). Decannulation and functional outcome after tracheostomy in patients with severe stroke (DECAST): A prospective observational study. *Neurocritical care,**27*(1), 26–34. 28324263 10.1007/s12028-017-0390-y

[6] Schroder, J. B., Marian, T., Muhle, P., Claus, I., Thomas, C., Ruck, T., Wiendl, H., Warnecke, T., Suntrup-Krueger, S., Meuth, S., et al. (2019). Intubation, tracheostomy, and decannulation in patients with Guillain-Barre-syndrome-does dysphagia matter? *Muscle & Nerve,**59*(2), 194–200. 30390307 10.1002/mus.26377

[7] Ponfick, M., Wiederer, R., Bösl, K., Neumann, G., Lüdemann-Podubecka, J., Gdynia, H. J., & Nowak, D. A. (2014). The influence of weaning duration on rehabilitative outcome in early neurological rehabilitation. *NeuroRehabilitation,**34*(3), 493–498. 24473250 10.3233/NRE-141066

[8] Pohl, M., Bertram, M., Bucka, C., Hartwich, M., Jöbges, M., Ketter, G., Leineweber, B., Mertl-Rötzer, M., Nowak, D., & Platz, T. (2016). Rehabilitationsverlauf von Patienten in der neurologisch-neurochirurgischen Frührehabilitation. *Der Nervenarzt,**87*(6), 634–644. 27090897 10.1007/s00115-016-0093-1

[9] Pohl, M., & Frührehabilitation, A.N.-N. (2017). Rehabilitationsverlauf von Patienten nach schwerem Schädel-Hirn-Trauma. *Neuroreha,**9*(03), 131–134.

[10] Heidler, M. D., Salzwedel, A., Jöbges, M., Lück, O., Dohle, C., Seifert, M., von Helden, A., Hollweg, W., & Völler, H. (2018). Decannulation of tracheotomized patients after long-term mechanical ventilation - results of a prospective multicentric study in German neurological early rehabilitation hospitals. *BMC anesthesiology,**18*(1), 65. 29898662 10.1186/s12871-018-0527-3 PMC6000940

[11] Röttinger, A., Seidel, G., Kücken, D., Zukunft, E., Töpper, R., Majewski, A., Klose, K., Terborg, C., Klass, I., & Debacher, U. (2018). Unterschiede im Verlauf der neurologischen Frührehabilitation bei Patienten nach Hirninfarkt, intrazerebraler Blutung und nicht-traumatischer Subarachnoidalblutung. *Aktuelle Neurologie,**45*(09), 646–654.

[12] Pohl, M., Berger, K., Ketter, G., Krusch, C., Pause, M., Puschendorf, W., Schaupp, M., Schleep, J., Spranger, M., & Steube, D. (2011). Langzeitverlauf von Patienten der neurologischen Rehabilitation Phase B. *Der Nervenarzt,**82*(6), 753–763. 20857274 10.1007/s00115-010-3119-0

[13] Hafner, G., Neuhuber, A., Hirtenfelder, S., Schmedler, B., & Eckel, H. E. (2008). Fiberoptic endoscopic evaluation of swallowing in intensive care unit patients. *European Archives of oto-Rhino-Laryngology Official Journal of the European Federation of Oto-Rhino-Laryngological Societies (EUFOS) : Affiliated with the German Society for Oto-Rhino-Laryngology - Head and Neck Surgery,**265*, 441–446. 17968575 10.1007/s00405-007-0507-6 PMC2254469

[14] Bosel, J. (2017). Use and timing of tracheostomy after severe stroke. *Stroke; a Journal of Cerebral Circulation,**48*(9), 2638–2643. 28733479 10.1161/STROKEAHA.117.017794

[15] Rollnik, J. D., Brocke, J., Gorsler, A., Groß, M., Hartwich, M., Pohl, M., Schmidt-Wilcke, T., & Platz, T. (2020). Weaning in der neurologisch-neurochirurgischen Frührehabilitation-Ergebnisse der „WennFrüh “-Studie der Deutschen Gesellschaft für Neurorehabilitation. *Der Nervenarzt,**91*(12), 1122–1129. 32776234 10.1007/s00115-020-00976-z PMC7416590

[16] Ledl, C., Frank, U., & Ullrich, Y. Y. (2023). Tracheostomy managment and tube weaning within a framework of dysphagia intervention. *Der Nervenarzt,**94*(8), 694–701. 37219566 10.1007/s00115-023-01489-1

[17] Ledl, C., Frank, U., Dziewas, R., Arnold, B., Bähre, N., Betz, C. S., Braune, S., Deitmer, T., Diesener, P., Fischer, A. S., et al. (2024). Curriculum “Tracheostomy management in dysphagia therapy.” *Der Nervenarzt,**95*(4), 342–352. 38277047 10.1007/s00115-023-01598-x PMC11014872

[18] Speed, L., & Harding, K. E. (2013). Tracheostomy teams reduce total tracheostomy time and increase speaking valve use: A systematic review and meta-analysis. *Journal of Critical Care,**28*(2), 216.e211–210. 10.1016/j.jcrc.2012.05.005 22951017

[19] Kutsukutsa, J., Kuupiel, D., Monori-Kiss, A., Del Rey-Puech, P., & Mashamba-Thompson, T. P. (2019). Tracheostomy decannulation methods and procedures for assessing readiness for decannulation in adults: A systematic scoping review. *International Journal of Evidence-Based Healthcare,**17*, 74. 31162271 10.1097/XEB.0000000000000166

[20] Welton, C., Morrison, M., Catalig, M., Chris, J., & Pataki, J. (2016). Can an interprofessional tracheostomy team improve weaning to decannulation times? A quality improvement evaluation. *Canadian Journal of Respiratory Therapy: CJRT = Revue canadienne de la therapie respiratoire : RCTR,**52*(1), 7–11. 26909008 PMC4751971

[21] Pandian, V., Miller, C. R., Schiavi, A. J., Yarmus, L., Contractor, A., Haut, E. R., Feller-Kopman, D. J., Mirski, M. A., Morad, A. H., Carey, J. P., et al. (2014). Utilization of a standardized tracheostomy capping and decannulation protocol to improve patient safety. *The Laryngoscope,**124*(8), 1794–1800. 24473939 10.1002/lary.24625

[22] Zivi, I., Valsecchi, R., Maestri, R., Maffia, S., Zarucchi, A., Molatore, K., Vellati, E., Saltuari, L., & Frazzitta, G. (2018). Early rehabilitation reduces time to decannulation in patients with severe acquired brain injury: A retrospective Study. *Frontiers in Neurology,**9*, 559. 30042728 10.3389/fneur.2018.00559 PMC6048253

[23] Frank, U., Mader, M., & Sticher, H. (2007). Dysphagic patients with tracheotomies: A multidisciplinary approach to treatment and decannulation management. *Dysphagia,**22*(1), 20–29. 17024547 10.1007/s00455-006-9036-5

[24] Bedwell, J. R., Pandian, V., Roberson, D. W., McGrath, B. A., Cameron, T. S., & Brenner, M. J. (2019). Multidisciplinary tracheostomy care: How collaboratives drive quality improvement. *Otolaryngologic Clinics of North America,**52*(1), 135–147. 30297183 10.1016/j.otc.2018.08.006

[25] Mah, J. W., Staff, I. I., Fisher, S. R., & Butler, K. L. (2017). Improving decannulation and swallowing function: A comprehensive, multidisciplinary approach to post-tracheostomy care. *Respiratory Care,**62*(2), 137–143. 28108683 10.4187/respcare.04878

[26] Farrell, M. S., Gillin, T. M., Emberger, J. S., Getchell, J., Caplan, R. J., Cipolle, M. D., & Bradley, K. M. (2019). Improving tracheostomy decannulation rate in trauma patients. *Critical Care Explorations,**1*(7), e0022. 31984377 10.1097/CCE.0000000000000022 PMC6980485

[27] Mussa, C. C., Gomaa, D., Rowley, D. D., Schmidt, U., Ginier, E., & Strickland, S. L. (2021). AARC clinical practice guideline: Management of adult patients with tracheostomy in the acute care setting. *Respiratory Care,**66*(1), 156–169. 32962998 10.4187/respcare.08206

[28] Likar, R., Aroyo, I., Bangert, K., Degen, B., Dziewas, R., Galvan, O., Grundschober, M.T., Köstenberger, M., Muhle, P., Schefold, J.C., & Zuercher, P. (2024). Management of swallowing disorders in ICU patients - A multinational expert opinion.* Journal of Critical Care, 79*, 154447. 10.1016/j.jcrc.2023.154447 37924574 10.1016/j.jcrc.2023.154447

[29] Dziewas, R., Allescher, H. D., Aroyo, I., Bartolome, G., Beilenhoff, U., Bohlender, J., Breitbach-Snowdon, H., Fheodoroff, K., Glahn, J., Heppner, H. J., et al. (2021). Diagnosis and treatment of neurogenic dysphagia - S1 guideline of the German society of neurology. *Neurological Research and Practice,**3*(1), 23. 33941289 10.1186/s42466-021-00122-3 PMC8094546

[30] Hales, P. A., Drinnan, M. J., & Wilson, J. A. (2008). The added value of fibreoptic endoscopic evaluation of swallowing in tracheostomy weaning. *Clinical Otolaryngology,**33*, 319–324. 18983340 10.1111/j.1749-4486.2008.01757.x

[31] Peruzzi, W. T., Logemann, J. A., Currie, D., & Moen, S. G. (2001). Assessment of aspiration in patients with tracheostomies: Comparison of the bedside colored dye assessment with videofluoroscopic examination. *Respiratory Care,**46*(3), 243–247. 11262550

[32] Donzelli, J., Brady, S., Wesling, M., & Craney, M. (2001). Simultaneous modified Evans blue dye procedure and video nasal endoscopic evaluation of the swallow. *The Laryngoscope,**111*, 1746–1750. 11801938 10.1097/00005537-200110000-00015

[33] Brady, S. L., Hildner, C. D., & Hutchins, B. F. (1999). Simultaneous videofluoroscopic swallow study and modified Evans blue dye procedure: An evaluation of blue dye visualization in cases of known aspiration. *Dysphagia,**14*(3), 146–149. 10341110 10.1007/PL00009596

[34] Linhares Filho, TA., Arcanjo, F. P. N., Zanin, L. H., Portela, H. A., Braga, J. M., da Luz Pereira, V. 2019. The accuracy of the modified Evan’ s blue dye test in detecting aspiration in tracheostomised patients. *The Journal of Laryngology and Otology* :1–4. 10.1017/S0022215119000471 30929652

[35] Bechet, S., Hill, F., Gilheaney, O., & Walshe, M. (2016). Diagnostic accuracy of the modified Evan’ s blue dye test in detecting aspiration in patients with tracheostomy: A systematic review of the evidence. *Dysphagia,**31*, 721. 27530728 10.1007/s00455-016-9737-3

[36] Belafsky, P., Blumenfeld, L., LePage, A., & Nahrstedt, K. (2003). The accuracy of the modified Evan’ s blue dye test in predicting aspiration. *The Laryngoscope,**113*, 1969–1972. 14603057 10.1097/00005537-200311000-00021

[37] Fiorelli, A., Ferraro, F., Nagar, F., Fusco, P., Mazzone, S., Costa, G., Di Natale, D., Serra, N., & Santini, M. (2017). A New modified Evans blue dye test as screening test for aspiration in tracheostomized patients. *Journal of cardiothoracic and vascular anesthesia,**31*(2), 441–445. 27720495 10.1053/j.jvca.2016.07.031

[38] Morris, K., Taylor, N. F., & Freeman-Sanderson, A. (2024). Safety-related outcomes for patients with a tracheostomy and the use of flexible endoscopic evaluation of swallowing (FEES) for assessment and management of swallowing: A systematic review. *International Journal of Speech-Language Pathology* :1–11. 10.1080/17549507.2023.2293633 38462820

[39] Warnecke, T., Labeit, B., Schroeder, J., Reckels, A., Ahring, S., Lapa, S., Claus, I., Muhle, P., Suntrup-Krueger, S., & Dziewas, R. (2021). Neurogenic dysphagia: Systematic review and proposal of a classification system. *Neurology,**96*(6), e876–e889. 33318164 10.1212/WNL.0000000000011350

[40] Dziewas, R., Warnecke, T., Labeit, B., Claus, I., Muhle, P., Oelenberg, S., Ahring, S., Wüller, C., Jung, A., von Itter, J., et al. (2024). Systematic approach to contextualize findings of flexible endoscopic evaluation of swallowing in neurogenic dysphagia- towards an integrated FEES report. *Neurological Research and Practice,**6*(1), 26. 38720388 10.1186/s42466-024-00321-8 PMC11080162

[41] Aviv, J. E., Kaplan, S. T., Thomson, J. E., Spitzer, J., Diamond, B., & Close, L. G. (2000). The safety of flexible endoscopic evaluation of swallowing with sensory testing (FEESST): An analysis of 500 consecutive evaluations. *Dysphagia,**15*(1), 39–44. 10594257 10.1007/s004559910008

[42] Aviv, J. E., Kaplan, S. T., Langmore, S. E. (2001). The safety of endoscopic swallowing evaluations. In: *Endoscopic evaluation and treatment of swallowing disorders.* edn. Edited by Langmore SE. New York, Stuttgart: Thieme, pp. 235–242.

[43] Cohen, M. A., Setzen, M., & Perlman, P. W. (2003). The safety of flexible endoscopic evaluation of swallowing with sensory testing in an outpatient otolaryngology setting. *The Laryngoscope,**113*, 21–24. 12514376 10.1097/00005537-200301000-00004

[44] Dziewas, R., Auf dem Brinke, M., Birkmann, U., Bräuer, G., Busch, K., Cerra, F., Damm-Lunau, R., Dunkel, J., Fellgiebel, A., Garms, E., & Glahn, J. (2019). Safety and clinical impact of FEES– results of the FEES-registry. *Neurological Practice and Research,**1*(1), 1–8. 10.1186/s42466-019-0021-5 PMC7650078 33324882

[45] Langmore, S. E. (2003). Evaluation of oropharyngeal dysphagia: Which diagnostic tool is superior? *Current Opinion in Otolaryngology & Head and Neck Surgery,**11*, 485–489. 14631184 10.1097/00020840-200312000-00014

[46] Aroyo, I., & Dziewas, R. (2022). Dysphagia in the intensive care unit – diagnosis and management (Dysphagie auf der Intensivstation – Diagnostik und Management). *DIVI,**2*, 74–79.

[47] Warnecke, T., Suntrup, S., Teismann, I. K., Hamacher, C., Oelenberg, S., & Dziewas, R. (2013). Standardized endoscopic swallowing evaluation for tracheostomy decannulation in critically ill neurologic patients. *Critical Care Medicine,**41*(7), 1728–1732. 23774336 10.1097/CCM.0b013e31828a4626

[48] Muhle, P., Suntrup-Krueger, S., Burkardt, K., Lapa, S., Ogawa, M., Claus, I., Labeit, B., Ahring, S., Oelenberg, S., Warnecke, T., et al. (2021). Standardized endoscopic swallowing evaluation for tracheostomy decannulation in critically ill neurologic patients - a prospective evaluation. *Neurological Research and Practice,**3*(1), 26. 33966636 10.1186/s42466-021-00124-1 PMC8108459

[49] Warnecke, T., Muhle, P., Claus, I., Schröder, J. B., Labeit, B., Lapa, S., Suntrup-Krueger, S., & Dziewas, R. (2020). Inter-rater and test-retest reliability of the “standardized endoscopic swallowing evaluation for tracheostomy decannulation in critically ill neurologic patients.” *Neurological Research and Practice,**2*, 1–7. 33324915 10.1186/s42466-020-00055-3 PMC7650070

[50] Bishnoi, T., Sahu, P. K., & Arjun, A. P. (2022). Evaluation of factors determining tracheostomy decannulation failure rate in adults: An Indian perspective descriptive study. *Indian Journal of Otolaryngology and Head and Neck Surgery: Official Publication of the Association of Otolaryngologists of India*, *74*(Suppl 3), 4849–4854. 10.1007/s12070-020-01982-y 32837943 10.1007/s12070-020-01982-y PMC7380661

[51] Meenan, K., Bhatnagar, K., & Guardiani, E. (2021). Intubation-related laryngeal pathology precluding tracheostomy decannulation: Incidence and associated risk factors. *The Annals of Otology, Rhinology, and Laryngology,**130*(9), 1078–1084. 33583187 10.1177/0003489421995285

[52] Thomas, A. J., Talbot, E., & Drewery, H. (2020). Failure of standard tracheostomy decannulation criteria to detect suprastomal pathology. *Anaesthesia Reports,**8*(1), 67–70. 33163965 10.1002/anr3.12048 PMC7605408

[53] Kutsukutsa, J., Kuupiel, D., Monori-Kiss, A., Del Rey-Puech, P., & Mashamba-Thompson, T. P. (2019). Tracheostomy decannulation methods and procedures for assessing readiness for decannulation in adults: A systematic scoping review. *International Journal of Evidence-Based Healthcare,**17*(2), 74–91. 31162271 10.1097/XEB.0000000000000166

[54] Bach, J. R., Saporito, L. R. (1994). Indications and criteria for decannulation and transition from invasive to noninvasive long-term ventilatory support. *Respiratory care* 3**9**(5):515–528; discussion 529–531. 10146010

[55] Chan, L. Y., Jones, A. Y., Chung, R. C., & Hung, K. N. (2010). Peak flow rate during induced cough: A predictor of successful decannulation of a tracheotomy tube in neurosurgical patients. *American Journal of Critical Care : An Official Publication, American Association of Critical-Care Nurses,**19*(3), 278–284. 19435950 10.4037/ajcc2009575

[56] Bach, J. R., & Saporito, L. R. (1996). Criteria for extubation and tracheostomy tube removal for patients with ventilatory failure. A different approach to weaning. *Chest,**110*(6), 1566–1571. 8989078 10.1378/chest.110.6.1566

[57] Khamiees, M., Raju, P., DeGirolamo, A., Amoateng-Adjepong, Y., & Manthous, C. A. (2001). Predictors of extubation outcome in patients who have successfully completed a spontaneous breathing trial. *Chest,**120*(4), 1262–1270. 11591570 10.1378/chest.120.4.1262

[58] Duan, J., Zhou, L., Xiao, M., Liu, J., & Yang, X. (2015). Semiquantitative cough strength score for predicting reintubation after planned extubation. *American Journal of Critical Care : An Official Publication, American Association of Critical-Care Nurses,**24*(6), e86-90. 26523016 10.4037/ajcc2015172

[59] Abedini, M., Froutan, R., Bagheri Moghaddam, A., & Mazloum, S. R. (2020). Comparison of “cough peak expiratory flow measurement” and “cough strength measurement using the white card test” in extubation success: A randomized controlled trial. *Journal of Research in Medical Sciences : The Official Journal of Isfahan University of Medical Sciences,**25*, 52. 32765622 10.4103/jrms.JRMS_939_19 PMC7377116

[60] Hernández, G., Ortiz, R., Pedrosa, A., Cuena, R., Vaquero Collado, C., González Arenas, P., García Plaza, S., Canabal Berlanga, A., & Fernández, R. (2012). The indication of tracheotomy conditions the predictors of time to decannulation in critical patients. *Medicina intensiva,**36*(8), 531–539. 22398327 10.1016/j.medin.2012.01.010

[61] Hernández Martínez, G., Rodriguez, M. L., Vaquero, M. C., Ortiz, R., Masclans, J. R., Roca, O., Colinas, L., de Pablo, R., Espinosa, M. D., Garcia-de-Acilu, M., et al. (2020). High-flow oxygen with capping or suctioning for tracheostomy decannulation. *The New England Journal of Medicine,**383*(11), 1009–1017. 32905673 10.1056/NEJMoa2010834

[62] Coplin, W. M., Pierson, D. J., Cooley, K. D., Newell, D. W., & Rubenfeld, G. D. (2000). Implications of extubation delay in brain-injured patients meeting standard weaning criteria. *American Journal of Respiratory and Critical Care Medicine,**161*(5), 1530–1536. 10806150 10.1164/ajrccm.161.5.9905102

[63] Steidl, C., Boesel, J., Suntrup-Krueger, S., Schoenenberger, S., Al-Suwaidan, F., Warnecke, T., Minnerup, J., & Dziewas, R. (2017). Tracheostomy, extubation, reintubation: Airway management decisions in intubated stroke patients. *Cerebrovascular Diseases (Basel, Switzerland),**44*(1–2), 1–9. 28395275 10.1159/000471892

[64] Suntrup-Krueger, S., Schmidt, S., Warnecke, T., Steidl, C., Muhle, P., Schroeder, J. B., Labeit, B., Minnerup, J., & Dziewas, R. (2019). Extubation readiness in critically ill stroke patients. *Stroke; a Journal of Cerebral Circulation,**50*(8), 1981–1988. 31280655 10.1161/STROKEAHA.118.024643

[65] Rumbak, M. J., Newton, M., Truncale, T., Schwartz, S. W., Adams, J. W., & Hazard, P. B. (2004). A prospective, randomized, study comparing early percutaneous dilational tracheotomy to prolonged translaryngeal intubation (delayed tracheotomy) in critically ill medical patients. *Critical Care Medicine,**32*(8), 1689–1694. 15286545 10.1097/01.ccm.0000134835.05161.b6

[66] Schönhofer, B., Geiseler, J., Dellweg, D., Fuchs, H., Moerer, O., Weber-Carstens, S., Westhoff, M., Windisch, W., Hirschfeld-Araujo, J., & Janssens, U. (2019). Prolongiertes weaning. *Pneumologie (Stuttgart, Germany),**73*(12), 723–814. 31816642 10.1055/a-1010-8764

[67] Johnson, D. C., Campbell, S. L., & Rabkin, J. D. (2009). Tracheostomy tube manometry: Evaluation of speaking valves, capping and need for downsizing. *The Clinical Respiratory Journal,**3*(1), 8–14. 20298366 10.1111/j.1752-699X.2008.00100.x

[68] Schwegler, H. (2016). Trachealkanülenmanagement: Dekanülierung beginnt auf der Intensivstation: Schulz-Kirchner Verlag GmbH

[69] Ceriana, P., Carlucci, A., Navalesi, P., Rampulla, C., Delmastro, M., Piaggi, G., De Mattia, E., & Nava, S. (2003). Weaning from tracheotomy in long-term mechanically ventilated patients: Feasibility of a decisional flowchart and clinical outcome. *Intensive Care Medicine,**29*(5), 845–848. 12634987 10.1007/s00134-003-1689-z

[70] Santus, P., Gramegna, A., Radovanovic, D., Raccanelli, R., Valenti, V., Rabbiosi, D., Vitacca, M., & Nava, S. (2014). A systematic review on tracheostomy decannulation: A proposal of a quantitative semiquantitative clinical score. *BMC Pulmonary Medicine,**14*, 201. 25510483 10.1186/1471-2466-14-201 PMC4277832

[71] Stelfox, H. T., Crimi, C., Berra, L., Noto, A., Schmidt, U., Bigatello, L. M., & Hess, D. (2008). Determinants of tracheostomy decannulation: An international survey. *Critical Care (London, England),**12*(1), R26. 18302759 10.1186/cc6802 PMC2374629

[72] Budweiser, S., Baur, T., Jörres, R. A., Kollert, F., Pfeifer, M., & Heinemann, F. (2012). Predictors of successful decannulation using a tracheostomy retainer in patients with prolonged weaning and persisting respiratory failure. *Respiration; International Review of Thoracic Diseases,**84*(6), 469–476. 22354154 10.1159/000335740

[73] Tobin, A. E., & Santamaria, J. D. (2008). An intensivist-led tracheostomy review team is associated with shorter decannulation time and length of stay: A prospective cohort study. *Critical Care (London, England),**12*(2), R48. 18402705 10.1186/cc6864 PMC2447599

[74] Enrichi, C., Battel, I., Zanetti, C., Koch, I., Ventura, L., Palmer, K., Meneghello, F., Piccione, F., Rossi, S., Lazzeri, M., et al. (2017). Clinical criteria for tracheostomy decannulation in subjects with acquired brain injury. *Respiratory Care,**62*(10), 1255–1263. 28698267 10.4187/respcare.05470

[75] Küchler, J., Wojak, J. F., Smith, E., Brocke, J., Abusamha, A., Tronnier, V. M., & Ditz, C. (2019). Management of tracheostomized patients after poor grade subarachnoid hemorrhage: Disease related and pulmonary risk factors for failed and delayed decannulation. *Clinical Neurology and Neurosurgery,**184*, 105419. 31306892 10.1016/j.clineuro.2019.105419

[76] Cohen, O., Tzelnick, S., Lahav, Y., Stavi, D., Shoffel-Havakuk, H., Hain, M., Halperin, D., & Adi, N. (2016). Feasibility of a single-stage tracheostomy decannulation protocol with endoscopy in adult patients. *The Laryngoscope,**126*(9), 2057–2062. 26607056 10.1002/lary.25800

[77] Schröter-Morasch, H. (2018). Medizinische Basisversorgung von Patienten mit Schluckstörungen–Trachealkanülen–Sondenernährung. In: *Schluckstörungen Diagnostik und Rehabilitation.* 6. edn. Edited by Bartolome G, Schröter-Morasch H. München: Elsevier, Urban&Fischer, pp. 215–260.

[78] O’ Connor, L. R., Morris, N. R., & Paratz, J. (2019). Physiological and clinical outcomes associated with use of one-way speaking valves on tracheostomised patients: A systematic review. *Heart & Lung : The Journal of Critical Care,**48*(4), 356–364. 30573194 10.1016/j.hrtlng.2018.11.006

[79] Ledl, C., & Ullrich, Y. Y. (2017). Occlusion of tracheostomy tubes does not alter pharyngeal phase kinematics but reduces penetration by enhancing pharyngeal clearance: A prospective study in patients with neurogenic dysphagia. *American Journal of Physical Medicine & Rehabilitation,**96*(4), 268–272. 27552353 10.1097/PHM.0000000000000602

[80] Pandian, V., Boisen, S., Mathews, S., & Brenner, M. J. (2019). Speech and safety in tracheostomy patients receiving mechanical ventilation: A systematic review. *American Journal of Critical Care : An Official Publication, American Association of Critical-Care Nurses,**28*(6), 441–450. 31676519 10.4037/ajcc2019892

[81] Medeiros, G. C., Sassi, F. C., Lirani-Silva, C., & Andrade, C. R. F. (2019). Criteria for tracheostomy decannulation: Literature review. *CoDAS,**31*(6), e20180228. 31800881 10.1590/2317-1782/20192018228

[82] Ross, J., McMurray, K., Cameron, T., & Lanteri, C. (2019). Use of a silicon stoma stent as an interim step in high-risk tracheostomy decannulation. *OTO Open,**3*(1), 2473974x19836432. 31236540 10.1177/2473974X19836432 PMC6572920

[83] Petosic, A., Viravong, M. F., Martin, A. M., Nilsen, C. B., Olafsen, K., & Berntzen, H. (2021). Above cuff vocalisation (ACV): A scoping review. *Acta anaesthesiologica Scandinavica,**65*(1), 15–25. 32920849 10.1111/aas.13706 PMC7756796

[84] Gajic, S., Jacobs, L., Gellentien, C., Dubin, R. M., & Ma, K. (2024). Implementation of above-cuff vocalization after tracheostomy is feasible and associated with earlier speech. *American Journal of Speech-Language Pathology / American Speech-Language-Hearing Association,**33*(1), 51–56. 10.1044/2023_AJSLP-23-00184 38056485

[85] Mills, C. S., Michou, E., King, N., Bellamy, M. C., Siddle, H. J., Brennan, C. A., & Bojke, C. (2022). Evidence for above cuff vocalization in patients with a tracheostomy: A systematic review. *The Laryngoscope,**132*(3), 600–611. 33932229 10.1002/lary.29591

[86] Ledl, C., Mertl-Rötzer, M., & Schaupp, M. (2016). Modernes Dysphagiemanagement in der neurologisch-neurochirurgischen Frührehabilitation. *Neurologie und Rehabilitation,**22*(3), 231–250.

[87] Bartolome, G. (2018). Funktionelle Dysphagietherapie bei speziellen neurologischen Erkrankungen. In: *Schluckstörungen Interdisziplinäre Diagnostik und Rehabilitation.* edn. Edited by Bartolome G, Schröter-Morasch H. München: Elsevier, Urban&Fischer, pp. 403–432.

[88] Dziewas, R., Pflug, C. (2020). Neurogene Dysphagie, S1-Leitliniein. In: *Leitlinien für Diagnostik und Therapie in der Neurologie.* edn. Edited by DGN;

[89] Benfield, J. K., Everton, L. F., Bath, P. M., & England, T. J. (2019). Does therapy with biofeedback improve swallowing in adults with dysphagia? A systematic review and meta-analysis. *Archives of Physical Medicine and Rehabilitation,**100*(3), 551–561. 29859178 10.1016/j.apmr.2018.04.031

[90] Kwong, E., Ng, K. K., Leung, M. T., & Zheng, Y. P. (2021). Application of ultrasound biofeedback to the learning of the mendelsohn maneuver in non-dysphagic adults: A pilot study. *Dysphagia,**36*(4), 650–658. 32889626 10.1007/s00455-020-10179-y

[91] Manor, Y., Mootanah, R., Freud, D., Giladi, N., & Cohen, J. T. (2013). Video-assisted swallowing therapy for patients with Parkinson’ s disease. *Parkinsonism & Related Disorders,**19*(2), 207–211. 23131836 10.1016/j.parkreldis.2012.10.004

[92] Langmore, S. E. (1996). Dysphagia in neurologic patients in the intensive care unit. *Seminars in Neurology,**16*, 329–340. 9112312 10.1055/s-2008-1040991

[93] Robbins, J., Gensler, G., Hind, J., Logemann, J. A., Lindblad, A. S., Brandt, D., Baum, H., Lilienfeld, D., Kosek, S., Lundy, D., et al. (2008). Comparison of 2 interventions for liquid aspiration on pneumonia incidence: A randomized trial. *Annals of Internal Medicine,**148*(7), 509–518. 18378947 10.7326/0003-4819-148-7-200804010-00007 PMC2364726

[94] Pisegna, J. M., & Langmore, S. E. (2018). The ice chip protocol: A description of the protocol and case reports. *Perspectives of the ASHA Special Interest Groups,**3*(13), 28–46.

[95] Pisegna, J. M., & Murray, J. (2018). Clinical application of flexible endoscopic evaluation of swallowing in stroke. *Seminars in Speech and Language,**39*(1), 3–14. 29359301 10.1055/s-0037-1608855

[96] Steffen, A., Jost, W., Bäumer, T., Beutner, D., Degenkolb-Weyers, S., Groß, M., Grosheva, M., Hakim, S., Kahl, K. G., Laskawi, R., et al. (2019). Hypersalivation - update of the S2k guideline (AWMF) in short form. *Laryngo- rhino- otologie,**98*(6), 388–397. 31167292 10.1055/a-0874-2406

[97] Chao, W., You-Qin, M., Hong, C., Hai-Ying, Z., Yang, L., Su-Xue, J., Lan, X., & Zhong, W. (2023). Effect of capsaicin atomization on cough and swallowing function in patients with hemorrhagic stroke: A randomized controlled trial. *Journal of Speech, Language, and Hearing Research : JSLHR,**66*(2), 503–512. 36716393 10.1044/2022_JSLHR-22-00296

[98] Wu, C., Zhang, Y., Yang, L., Shen, F., Ma, C., & Shen, M. (2021). Effect of capsaicin atomization-induced cough on sputum excretion in tracheotomized patients after hemorrhagic stroke: A randomized controlled trial. *Journal of Speech, Language, and Hearing Research : JSLHR,**64*(11), 4085–4095. 34694869 10.1044/2021_JSLHR-21-00151

[99] Lüthi-Müller, E., Kool, J., Mylius, V., & Diesener, P. (2022). A new therapeutic approach for dystussia and atussia in neurogenic dysphagia: Effect of aerosolized capsaicin on peak cough flow. *Dysphagia,**37*(6), 1814–1821. 35430718 10.1007/s00455-022-10439-z PMC9643184

[100] Hamdy, S., Rothwell, J. C., Aziz, Q., Singh, K. D., & Thompson, D. G. (1998). Long-term reorganization of human motor cortex driven by short-term sensory stimulation. *Nature Neuroscience,**1*(1), 64–68. 10195111 10.1038/264

[101] Suntrup, S., Teismann, I., Wollbrink, A., Winkels, M., Warnecke, T., Pantev, C., & Dziewas, R. (2015). Pharyngeal electrical stimulation can modulate swallowing in cortical processing and behavior - magnetoencephalographic evidence. *NeuroImage,**104*, 117–124. 25451471 10.1016/j.neuroimage.2014.10.016

[102] Muhle, P., Labeit, B., Wollbrink, A., Claus, I., Warnecke, T., Wolters, C. H., Gross, J., Dziewas, R., & Suntrup-Krueger, S. (2021). Targeting the sensory feedback within the swallowing network-reversing artificially induced pharyngolaryngeal hypesthesia by central and peripheral stimulation strategies. *Human Brain Mapping,**42*(2), 427–438. 33068056 10.1002/hbm.25233 PMC7776007

[103] Muhle, P., Suntrup-Krueger, S., Bittner, S., Ruck, T., Claus, I., Marian, T., Schroder, J. B., Minnerup, J., Warnecke, T., Meuth, S. G., et al. (2017). Increase of substance P concentration in saliva after pharyngeal electrical stimulation in severely dysphagic stroke patients - an indicator of decannulation success? *Neuro-Signals,**25*(1), 74–87. 29041008 10.1159/000482002

[104] Muhle, P., Suntrup-Krueger, S., & Dziewas, R. (2018). Neurophysiological adaptation and neuromodulatory treatment approaches in patients suffering from post-stroke dysphagia. *Current Physical Medicine and Rehabilitation Reports,**6*(4), 227–238.

[105] Suntrup-Krueger, S., Bittner, S., Recker, S., Meuth, S. G., Warnecke, T., Suttrup, I., Marian, T., & Dziewas, R. (2016). Electrical pharyngeal stimulation increases substance P level in saliva. *Neurogastroenterology and Motility : The Official Journal of the European Gastrointestinal Motility Society,**28*(6), 855–860. 10.1111/nmo.12783 26871730

[106] Dziewas, R., Stellato, R., van der Tweel, I., Walther, E., Werner, C. J., Braun, T., Citerio, G., Jandl, M., Friedrichs, M., Notzel, K., et al. (2018). Pharyngeal electrical stimulation for early decannulation in tracheotomised patients with neurogenic dysphagia after stroke (PHAST-TRAC): A prospective, single-blinded, randomised trial. *The Lancet Neurology,**17*(10), 849–859. 30170898 10.1016/S1474-4422(18)30255-2

[107] Suntrup, S., Marian, T., Schroder, J. B., Suttrup, I., Muhle, P., Oelenberg, S., Hamacher, C., Minnerup, J., Warnecke, T., & Dziewas, R. (2015). Electrical pharyngeal stimulation for dysphagia treatment in tracheotomized stroke patients: A randomized controlled trial. *Intensive Care Medicine,**41*(9), 1629–1637. 26077087 10.1007/s00134-015-3897-8

[108] Bath, P. M., Woodhouse, L. J., Suntrup-Krueger, S., Likar, R., Koestenberger, M., Warusevitane, A., Herzog, J., Schuttler, M., Ragab, S., Everton, L., et al. (2020). Pharyngeal electrical stimulation for neurogenic dysphagia following stroke, traumatic brain injury or other causes: Main results from the PHADER cohort study. *EClinicalMedicine,**28*, 100608. 33294818 10.1016/j.eclinm.2020.100608 PMC7700977

[109] Cheng, I., Bath, P. M., Hamdy, S., Muhle, P., Mistry, S., Dziewas, R., & Suntrup-Krueger, S. (2024). Predictors of pharyngeal electrical stimulation treatment success in tracheotomised stroke patients with dysphagia: Secondary analysis from PHADER cohort study. *Neurotherapeutics : The Journal of the American Society for Experimental NeuroTherapeutics,**21*(5), e00433. 39181859 10.1016/j.neurot.2024.e00433 PMC11579862

[110] Koestenberger, M., Neuwersch, S., Hoefner, E., Breschan, C., Weissmann, H., Stettner, H., & Likar, R. (2019). A pilot study of pharyngeal electrical stimulation for orally intubated ICU patients with dysphagia. *Neurocritical Care,**32*, 532. 10.1007/s12028-019-00780-x 31313142

[111] Traugott, M., Hoepler, W., Kitzberger, R., Pavlata, S., Seitz, T., Baumgartner, S., Placher-Sorko, G., Pirker-Krassnig, D., Ehehalt, U., Grasnek, A., et al. (2021). Successful treatment of intubation-induced severe neurogenic post-extubation dysphagia using pharyngeal electrical stimulation in a COVID-19 survivor: A case report. *Journal of Medical Case Reports,**15*(1), 148. 33752743 10.1186/s13256-021-02763-z PMC7983095

[112] Traugott, M., Hoepler, W., Kelani, H., Schatzl, M., Friese, E., & Neuhold, S. (2022). Pharyngeal electrical stimulation treatment of critically Ill intensive care tracheostomized patients presenting with severe neurogenic dysphagia: A case series. *Austin J Pulm Respir Med,**9*(1), 1088.

[113] Florea, C., Bräumann, C., Mussger, C., Leis, S., Hauer, L., Sellner, J., & Golaszewski, S. M. (2020). Therapy of dysphagia by prolonged pharyngeal electrical stimulation (Phagenyx) in a patient with brainstem infarction. *Brain Sciences,**10*(5), 256. 32353976 10.3390/brainsci10050256 PMC7287930

[114] Suntrup-Krueger, S., Labeit, B., Marian, T., Schröder, J., Claus, I., Ahring, S., Warnecke, T., Dziewas, R., & Muhle, P. (2023). Pharyngeal electrical stimulation for postextubation dysphagia in acute stroke: A randomized controlled pilot trial. *Critical Care (London, England),**27*(1), 383. 37789340 10.1186/s13054-023-04665-6 PMC10548555

[115] Muhle, P., Claus, I., Labeit, B., Roderigo, M., Warnecke, T., Dziewas, R., & Suntrup-Krueger, S. (2024). Pharyngeal Electrical Stimulation prior to extubation - reduction of extubation failure rate in acute stroke patients? *Journal of Critical Care,**82*, 154808. 38581884 10.1016/j.jcrc.2024.154808

[116] Beirer, S., Grisold, W., & Dreisbach, J. (2020). Therapy-resistant dysphagia successfully treated using pharyngeal electrical stimulation in a patient with the pharyngeal-cervical-brachial variant of the Guillain-Barré syndrome. *eNeurologicalSci,**20*, 100255. 32715111 10.1016/j.ensci.2020.100255 PMC7372150

[117] Blakemore, C., Hunter, J., & Basu, B. (2021). Rapid swallow improvement following pharyngeal electrical stimulation in a COVID-19 patient with long-term severe neurogenic dysphagia: A case report. *Journal of Rehabilitation Medicine,**4*, jrmcc00075.

[118] Williams, T., Walkden, E., Patel, K., Cochrane, N. E., McGrath, B. A., & Wallace, S. (2024). Research report: Management of dysphagia using pharyngeal electrical stimulation in the general intensive care population - a service development. *Journal of the Intensive Care Society,**25*(4), 374–382. 39524066 10.1177/17511437241270244 PMC11549722

[119] Ledl, C., & Wagner-Sonntag, E. (2016). Highway to decanulation: A bi-center analysis of decanulation rates and causes of weaning failure. *Dysphagia,**31*, 270.

[120] Epstein, S. K. (2005). Late complications of tracheostomy. *Respiratory Care,**50*(4), 542–549. 15807919

[121] Chang, C. W. D., Mccoul, E. D., Briggs, S. E., Guardiani, E. A., Durand, M. L., Hadlock, T. A., Hillel, A. T., Kattar, N., Openshaw, P. J. M., Osazuwa-Peters, N., & Poetker, D. M. (2022). Corticosteroid use in otolaryngology current considerations during the COVID-19 era. *Otolaryngology Head and Neck Surgery: Official Journal of American Academy of Otolaryngology-Head and Neck Surgery,**167*(5), 803–820. 34874793 10.1177/01945998211064275

[122] Crane, J., Endo, T., & Fox, M. (2025). Tracheal resection for post-intubation/post-tracheostomy tracheal stenosis. *Thoracic Surgery clinics,**35*(1), 61–72. 39515896 10.1016/j.thorsurg.2024.09.001

